# Restless Legs Syndrome in Chinese Patients With Sporadic Amyotrophic Lateral Sclerosis

**DOI:** 10.3389/fneur.2018.00735

**Published:** 2018-08-30

**Authors:** Shuangwu Liu, Dongchao Shen, Hongfei Tai, Ning Su, Qingyun Ding, Hanhui Fu, Kang Zhang, Zhili Wang, Mingsheng Liu, Yan Huang, Liying Cui

**Affiliations:** ^1^Department of Neurology, Peking Union Medical College Hospital, Beijing, China; ^2^Neuroscience Center, Chinese Academy of Medical Sciences, Beijing, China

**Keywords:** amyotrophic lateral sclerosis, restless legs syndrome, excessive daytime sleepiness, sleep disorders, neurodegenerative disease

## Abstract

**Objective:** To evaluate the frequency and clinical features of restless legs syndrome (RLS) in a group of Chinese patients with amyotrophic lateral sclerosis (ALS).

**Methods:** 109 Patients included in this study fulfilled the revised El Escorial diagnostic criteria for clinically definite, probable and lab-supported probable ALS, and a group of 109 control subjects was matched for age and sex to the ALS group. Disease severity was assessed by the revised ALS functional rating scale (ALSFRS-R). The diagnosis of RLS was made according to the criteria of the International RLS Study Group. Other characteristics including sleep quality, excessive daytime sleepiness (EDS), REM sleep behavior disorder (RBD), depression and anxiety were also evaluated in ALS patients.

**Results:** RLS was significantly more frequent in ALS patients than in control subjects (14.6 vs. 0.9%; *P* < 0.05). Compared to those without RLS, ALS patients with RLS reported a higher frequency of anxiety and EDS. ALS patients with RLS showed more severe legs dysfunction. EDS and legs function scores of the ALSFRS-R were independent factors significantly associated with RLS in ALS patients.

**Conclusions:** Our findings suggest that Chinese ALS patients exhibit a high frequency of RLS symptoms and that these patients may benefit from recognition of the condition and optimized management of its symptoms. Moreover, ALS patients might cause circadian rhythms disturbance and our study further supports that ALS is a heterogeneous disorder involving multiple systems; further studies are needed to confirm these preliminary findings.

## Introduction

Amyotrophic lateral sclerosis (ALS) is a rare neurodegenerative disease with clinical and hereditary heterogeneity (1). While ALS is sporadic in most cases, 10% of patients are transmitted within families (1). The etiology of ALS is still unknown; however, the interactions between genetic background and environmental exposure are likely to underpin disease susceptibility (1–3). Although predominantly manifested by progressive muscle weakness, increasing evidence has demonstrated the non-pyramidal features of ALS including cognitive impairment, abnormal behavior and sleep disorders (3–5). Neuroimaging and pathological studies of ALS have shown that its pathophysiology is not limited to the motor cortex but also involves other cortical and subcortical structures (2, 3). Such diverse structures are likely the anatomical substrates of the clinical heterogeneity of ALS (3). Moreover, ALS patients of different races and from different areas seem to have different epidemiological characteristics (1, 6). Death typically occurs 3–5 years after diagnosis, although some forms of the disease permit protracted survival (1).

Restless legs syndrome (RLS) is a common neurological disorder that causes patients irresistible urge to move the legs, usually accompanied by an uncomfortable or unpleasant sensation (7, 8). The etiology of RLS remain poorly understood. RLS is circadian in nature and commonly causing major symptoms in the evening or at night (8, 9). RLS symptoms severely affect 1–5% of adults in general populations (9). At present, two different phenotypes of RLS are recognized (8). Primary RLS exhibits a peak onset at approximately 20–40 years of age, with slow disease evolution and frequently a family history of RLS (9). In contrast, late-onset RLS exhibits a peak onset after 40 years of age, less frequently with a family history of RLS and more frequent correlation with other conditions (10, 11). Increased frequency of late-onset RLS has been demonstrated in many patients with neurodegenerative disorders, such as Alzheimer's disease (AD) and Parkinson's disease (PD) (11–14). Two recent studies found a significantly higher frequency of RLS disturbances in Caucasian patients with ALS (12, 13). However, Panda et al reported a less frequency of RLS in Indian ALS patients, the conclusions of these studies were not completely consistent (15). To date, data on Chinese ALS patients are limited. Hence, we conducted a cross-sectional study to investigate the frequency and clinical features of RLS in a Chinese ALS cohort from our ALS center.

## Materials and methods

### Participants

ALS patients who visit to our ALS Center had been enrolled in our ongoing registry platform for motor neuron disease (MND) (6). The patients underwent a systematic medical history evaluation and a neurological examination. Demographic and clinical information, including age, gender, level of education, site of symptom onset, forced vital capacity (FVC), and disease duration (defined as the time between symptom onset and time of diagnosis), was collected. The revised ALS functional rating scale (ALSFRS-R) was used to assess disease severity and bulbar function was assessed by the swallowing subscale of the Amyotrophic Lateral Sclerosis Severity Scale (ALSSS) (16). Because RLS symptoms mainly involve the lower limbs, we added the walking subscale and the climbing stairs subscale of the ALSFRS-R to further analyze the legs function of ALS patients (13).

ALS patients included in this study fulfilled the revised El Escorial diagnostic criteria for clinically definite, probable and lab-supported probable ALS (17). The exclusion criteria included family history of ALS, inability to communicate, refusal to participate in the study.

Healthy controls (HCs) were all recruited from the family members of ALS patients and matched for age and sex to the ALS group.

This study was approved by the Research Ethics Committee of Peking Union Medical College Hospital. Participant information was collected only after all patients and HCs were aware of the purpose of this study and had written an informed consent letter.

### RLS diagnosis and assessment

The diagnosis of RLS was made using the four standard diagnostic criteria developed by the International Restless Leg Syndrome Study Group (IRLSSG) (18, 19). In 2012, the IRLSSG added a fifth criterion to improve the sensitivity of diagnosis: that the occurrences of the RLS symptoms are not solely accounted for as primary symptoms of another medical or behavioral condition (e.g., myalgia, venous stasis, leg edema, arthritis, leg cramps, positional discomfort, or habitual foot tapping) (19). Based on the new criterion and on the experience of our clinical practice of ALS, an additional questionnaire was designed for ALS patients to further exclude clinical conditions that could mimic RLS symptoms (including pain, nocturnal leg cramps, stiffness, and discomfort from spasticity or difficulty turning in bed) (13, 18). A positive diagnosis of RLS was made when a patient had all four symptoms described above and the mimicking symptoms could be excluded.

When patients received a diagnosis of RLS, information about their family history of RLS, drug intake (especially hypnotics and antidepressants) and past medical history was collected and laboratory tests (serum iron and ferritin) were also performed to exclude other, secondary forms of RLS.

RLS patients in the ALS and control groups who used dopamine antagonists or antidepressants or who had a family history of RLS, iron deficiency, renal insufficiency or a history of other neurological conditions that could induce secondary RLS were also excluded from our analysis (12, 13).

Subjects in each group (both ALS and health control) were then divided into RLS+ and RLS– subgroups according to whether or not they had RLS. In addition, we also recorded the main clinical symptoms of RLS in patients with ALS.

### Sleep quality, excessive daytime sleepiness (EDS) and REM sleep behaviour disorder (RBD) assessments

Interviews regarding sleep disorders were conducted in ALS patients by the same investigator. Sleep quality was assessed using the Pittsburgh Sleep Quality Index (PSQI) (19). A PSQI score >5 indicates poor sleep (20). Excessive daytime sleepiness (EDS) was assessed using the Epworth Sleepiness Scale (ESS) (21). EDS was defined by an ESS score of ≥10 (21). RBD was assessed by the RBD screening questionnaire (RBDSQ), and RBD was indicated by total RBDSQ scores ≥5 (22).

### Anxiety and depression assessments

The diagnosis of depression and anxiety in ALS patients was established using the Hamilton Depression Rating Scale (HDRS) and the Hamilton Anxiety Rating Scale (HARS), respectively (23). The HDRS includes 17 items. Symptom severity was rated from 0 (not present) to 4 (very severe), and the scores ranged from 0 to 68; a score of >7 indicated depression. The HARS includes 14 items. Symptom severity was rated from 0 (not present) to 4 (very severe), and the scores ranged from 0 to 56; a score of >7 indicated anxiety (23).

### Polysomnography

Some patients with ALS were also assessed using overnight polysomnography in our sleep laboratory. The periodic limb movements in sleep (PLMS), apnoea/hypopnea index (AHI) and mean SaO_2_ overnight data were measured by visual inspection, and nocturnal pulse oximetry (NPO) was considered altered in accordance with the same methodology described in a previous study (5).

### Statistical analysis

Descriptive data of the population are expressed as number of cases and proportions for categorical variables, and as mean and SD for continuous variables. The Student's *t*-test (with Mann-Whitney U test when necessary) was used to compare continuous variables and the chi-square test was used to analyze categorical variables. The binary logistic regression model was used to evaluate the association between ALS and RLS. The risk factors for RLS in the ALS population were investigated by univariate statistical analysis and the results were controlled using a binary logistic regression model. Statistical significance was considered as a *p*-value < 0.05. All statistical analyses were performed using SPSS version 20.0 (IBM Corp., Armonk, NY).

## Results

### Demographic and clinical information of ALS patients

A total of 138 consecutive ALS patients were included in the study during their routine visits to our ALS Center between March 2017 and December 2017. Twenty-nine ALS patients were excluded (inability to communicate, *n* = 5; refusal, *n* = 3; ALS family history, *n* = 4; possible ALS, *n* = 17). Of the 109 remaining patients (43 women, 66 men), the mean age was 52.9 ± 10.1 years, and the mean disease duration was 15.6 ± 9.6 months. Thirty-six patients (33.0%) had bulbar onset of the disease. One patient was using non-invasive ventilation. One patient was receiving enteral nutrition. Forty-four patients (40.3%) were taking riluzole. A group of 109 HC subjects (48 women, 61 men; mean age 51.6 ± 11.4 years) was also included in the study. ALS Patients had a mean ALSFRS-R score of 40.2 ± 4.2, a mean ALSSS swallowing score of 10.3 ± 1.7, and a mean ALSFRS-R lower limb function score of 6.3 ± 1.0. A total of 80 ALS patients completed the FVC examination and a mean FVC of 90.9 ± 17.2. The FVC examination was not performed on the remaining 29 ALS patients because they had seriously physical disability and refused to perform the examination. The mean global PSQI score of ALS patients was 7.5 ± 4.4. The mean ESS scores of ALS patients was 5.72 ± 3.93. The mean RBDSQ score of ALS patients was 2.66 ± 2.03. The mean HARS scores of ALS patients was 5.77 ± 4.80, and the mean HDRS scores of ALS patients was 7.16 ± 4.94. The main demographic and clinical features of ALS patients are shown in Table 1.

**Table 1:** Demographic and clinical features of ALS patients.

	***N* = 109**
Age(years)	52.9 ± 10.1
Sex, *n* (%)	
Male	66 (60.6%)
Female	43 (39.4%)
Duration of ALS symptoms (mo)	15.6 ± 9.7
Bulbar onset, *n* (%)	36 (33.0%)
ALSFRS-R score	40.2 ± 4.2
ALSSS	10.3 ± 1.7
Poor sleeper, *n* (%)	68 (62.3%)
EDS, *n* (%)	17 (15.6%)
RBD, *n* (%)	11 (10.1%)
Anxiety, *n* (%)	29 (16.6%)
Depression, *n* (%)	43 (39.4%)
Riluzole, *n* (%)	44 (40.3%)

*ALS, Amyotrophic lateral sclerosis; ALSFRS-R, revised ALS functional rating scale; ALSSS, Amyotrophic Lateral Sclerosis Severity Scale; EDS, excessive daytime sleepiness; RBD, REM sleep behavior disorder. Values of continuous variables are reported as mean value ± standard deviation and categorical variables are reported as number of cases and proportions*.

### Frequency and clinical feature of RLS

A diagnosis of RLS was made in 16 (14.7%) patients with ALS (ALS/RLS+ group) and in 1 (0.9%) control subject (*p* < 0.05). No patients with RLS in the ALS and HC groups had a family history of RLS, had used antidepressants or had abnormal laboratory test results (all subjects serum ferritin level >50 ng/ml) (8). One ALS/RLS+ patient reported that RLS symptoms accompanied ALS onset, while the remaining ALS/RLS+ patients reported that RLS symptoms followed the onset of ALS. In the ALS/RLS+ group, six patients reported the frequency of RLS symptoms were once a week, five patients were two to three times a week, four patients were four to five times a week and two patients were six to seven times a week, no patient reported less once a week. Fifteen ALS/RLS+ patients reported the symptoms of RLS only affected the legs. ALS/RLS+ patients described the major RLS symptoms as an urge to move the legs that was commonly accompanied by uncomfortable feelings (58.9%), pain (17.6%), “burning” sensations (11.8%), and in 2 patients (11.8%), solely the urge to move without other symptoms. Compared with those without RLS (ALS/RLS– patients), ALS/RLS+ patients showed decreased legs function scores of the ALSFRS-R (*p* < 0.05), and other subscales of the ALSFRS-R and the ALSSS scores were not different between the two groups. The main demographic and clinical features of the ALS/RLS+ and ALS/RLS– groups are shown in Table 2.

**Table 2:** Characteristics of the patients with ALS with and without RLS.

	**ALS/RLS+ patients (*n* = 16)**	**ALS/RLS– patients (*n* = 93)**	***P*-value**
Age (years)	54.81 ± 7.32	52.58 ± 10.53	0.42
Men/Women (*n*)	8/8	58/35	0.35
Duration of ALS symptom (mo)	13.2 ± 5.9	16.0 ± 10.1	0.28
Bulbar onset, *n* (%)	6 (37.5)	30 (32.3)	0.68
ALSFRS-R score	38.9 ± 3.6	40.4 ± 4.3	0.18
Legs function	5.0 ± 1.7	6.6 ± 1.7	<0.01^**^
Walking	3.0 ± 0.5	3.5 ± 0.6	<0.01^**^
Climbing stairs	2.0 ± 1.2	3.1 ± 1.1	<0.01^**^
PSQI	9.4 ± 3.6	7.2 ± 4.5	0.06
Sleep quality	1.4 ± 0.7	1.1 ± 0.8	0.09
Sleep latency	2.2 ± 0.8	1.3 ± 0.9	<0.01^**^
sleep duration	1.4 ± 1.1	1.0 ± 1.0	0.24
Sleep efficiency	1.4 ± 1.0	1.3 ± 1.1	0.44
Sleep disturbances	1.4 ± 0.6	1.2 ± 0.5	0.26
Use of hypnotics	0.3 ± 0.9	0.2 ± 0.7	0.52
Daytime dysfunction	1.3 ± 1.1	1.1 ± 1.1	0.38
Poor sleeper *n*, (%)	11 (68.7%)	57 (61.2%)	0.48
ESS	8.3 ± 3.8	5.3 ± 3.8	<0.01^**^
EDS, *n* (%)	7 (43.75)	10 (10.75)	<0.01^**^
HARS	8.1 ± 6.0	5.1 ± 4.8	<0.05^*^
Anxiety, *n* (%)	8 (50.0%)	21 (22.5%)	<0.05^*^
HDRS	9.1 ± 5.3	6.8 ± 4.8	0.09
Depression	8 (50.0%)	35 (37.6%)	0.35
Riluzole, n (%)	6 (37.5%)	38 (40.8%)	0.80

*ALS, Amyotrophic lateral sclerosis; RLS, Restless legs syndrome; ALSFRS-R, revised ALS functional rating scale; PSQI, Pittsburgh sleep quality index; ESS, Epworth Sleepiness Scale; EDS, excessive daytime sleepiness; HARS, Hamilton Anxiety Rating Scale; HDRS, Hamilton Depression Rating Scale. Values of continuous variables are reported as mean value ± standard deviation and categorical variables are reported as number of cases and proportions*.

* p < 0.05;

** *p < 0.01*.

The logistic analyses that included age and sex as confounding factors confirmed an association between ALS and RLS and quantified the risk of RLS to be 19 (95% CI: 2.48–147.91, *p* < 0.01) times greater for ALS patients than for healthy subjects. Logistic regression analyses including age, sex, duration of ALS symptoms, HAMA scores, ALSFRS-R lower limb function scores and EDS as confounding factors, showed that ALSFRS-R legs function scores and EDS were independent factors significantly associated with RLS occurrence. In contrast, the independent effect of HAMA scores was lost. The results of the logistic regression analyses of the risk factors for RLS in ALS patients are presented in Table 3.

**Table 3:** Odds ratios of risk factors for RLS in ALS patients, by logistic regression analysis.

**Variables**	**OR (95% CI)**	***P*-value**
Age	1.06 (0.99–1.15)	0.13
Sex	0.83 (0.22–3.13)	0.79
Duration of RLS symptoms	0.93 (0.85–1.02)	0.08
ALSFRS-R leg function scores	0.61 (0.418–0.88)	0.01^*^
HARS	1.01 (0.89–1.14)	0.92
EDS	9.49 (1.91–47.29)	<0.01^**^

*RLS, Restless legs syndrome; ALS, Amyotrophic lateral sclerosis; OR, odds ratio; ALSFRS-R, revised ALS functional rating scale; EDS, excessive daytime sleepiness; HARS, Hamilton Anxiety Rating Scale*.

* p < 0.05;

** *p < 0.01*.

### Polysomnography

Ultimately, 12 patients with ALS underwent polysomnography and none had altered NPO. One of the 12 ALS patients showed RLS symptoms and recorded a PLMS index of ≥15 (16.5), giving further support to the RLS diagnosis. Four of the 12 ALS patients showed EDS symptoms but their recorded AHI (5.05 ± 3.96 vs. 5.98 ± 5.05, *p* > 0.05) and SaO_2_ (96.25 ± 1.25 vs. 96.14 ± 0.69, *p* > 0.05) data were not different to ALS patients without EDS.

## Discussion

This is the first study to investigate the frequency and clinical features of RLS in Chinese ALS patients. To our knowledge, this study is also the largest to investigate the association between RLS and ALS. Although this study did not use a population-based design, it adds credibility to descriptions of RLS in ALS patients due to its relatively large sample size.

Consistent with previous studies, we observed a significantly higher frequency of RLS symptoms in ALS patients (14.7%) than in control subjects (0.9%) or in the general population (9). Patients with ALS had a 19-fold increased risk of RLS than control subjects. Our observation also supports a secondary RLS form in ALS patients (13). However, many of our findings differ from those of previous cohorts. Our study showed that EDS and the leg function scores of the ALSFRS-R were independent factors significantly associated with RLS occurrence in ALS patients. Lo Coco D et al. reported that the total ALSFRS-R score was the only independent factor significantly associated with RLS, but they did not further examine the subscale scores of the ALSFRS-R (13). Compared with our study, the study by Lo Coco D et al. had a higher frequency of RLS symptom in ALS patients (25 vs. 14.6%) and these patients exhibited increased functional disability. Moreover, their patient group had a lower frequency of bulbar onset (13). Limousin et al. reported a higher frequency of RLS in a cohort of French ALS patients who were aged >64 years; they found that difficulty turning in bed and adjusting bedclothes was associated with RLS in ALS patients. However, compared with those in our study, their patients were older and had lower ESS scores (12) Furthermore, their ALS patients had longer disease courses. Neither of these studies examined anxiety or assessed sleep quality with the PSQI (12, 13).

Although an increasing number of studies have been performed that have enabled better understanding of some aspects of RLS, the cause and the anatomical origin remain unknown. Among the candidate causes, genetic predisposition, thalamic abnormalities, alteration of iron metabolism, and dysfunction of the dopaminergic system in the brain and spinal cord have been demonstrated and seem to be largely interconnected (8). Recently, abnormal circadian patterns associated with thalamic abnormalities in RLS patients have been demonstrated, and motor symptoms of RLS seem to be primarily produced by the dopaminergic system (24). Interestingly, in addition to ALS, other neurodegenerative diseases such as Alzheimer's disease or Parkinson's disease, which can induce secondary RLS, also seem to involve parts of the network described above (10, 11).

Although some ALS patients were bulbar onset, almost all patients have spinal cord dysfunction (1). Previous studies have suggested a major role of the spinal cord in RLS (18). We observed that ALS/RLS+ patients had more severe leg dysfunction. Dopamine acting in the spinal cord could modulate sensory and motor functions and might involve part of the RLS pathway. Dopamine receptors D1 to D5 are widely expressed in the mouse spinal cord, including in motoneurons and dorsal interneurons (25). Moreover, diseases that involve corticospinal tracts and are associated with development of RLS symptoms have been well-described (12). Furthermore, PLMS frequently accompanies spinal cord lesions and is found in nearly 80% of RLS patients, further supporting a role of the spinal cord in the condition (8). A progressive dysfunction of the spinal cord in ALS patients might explain the leg dysfunction associated with RLS occurrence in our study.

In our study, 17 patients (15.5%) with ALS had EDS, of which 7 were in the ALS/RLS+ group (43.8%), higher than in the ALS/RLS- group (10.8%). Consistent with our study, Limousin et al also reported a higher frequency of EDS symptoms in ALS patients with RLS (12). However, they did not discuss the correlation between EDS and RLS in ALS patients (12). Our observations further suggest that EDS is associated with RLS symptoms in ALS patients. Data on EDS in ALS patients are rather limited. Abnormal sleep-wake patterns and respiratory issues seem to contribute to the occurrence of EDS symptoms in ALS patients (5, 7). In the present study, because of a relatively shorter disease course, the ALSFRS-R respiratory scores were almost normal in ALS patients with EDS and only three patients were affected. Moreover, AHI and NPO were also not different in ALS patients with and without EDS who underwent polysomnography. Therefore, our study suggest that EDS symptoms were not completely due to respiratory disturbance in ALS patients (5).

EDS is a sleep-wake problem and patients with EDS symptoms may have thalamic abnormalities (26–28). Similar to EDS symptoms, RLS symptoms are circadian in nature which likely due to thalamic disturbances (29). Recently, an MR spectroscopy study further suggested that increased activation of the thalamic glutamatergic system was linked to the circadian disturbance in RLS (24). In fact, the role of the thalamus in regulating sleep-wake patterns may have been underestimated. Thalamic cellular and network mechanisms possibly underlie both the central and non-central regulation of the sleep-wake cycle (30). Several studies have shown that the substantia nigra and thalamus are affected in patients with ALS, but these studies did not further demonstrate whether there were circadian rhythm disturbances in these patients (31). Accordingly, as a disease of unknown origin with clinical and anatomical heterogeneity, we have demonstrated that a potential circadian rhythm disturbance, possibly due to thalamic abnormalities, exists in a group of Chinese patients with ALS. Further studies are needed to confirm these preliminary findings.

Conditions that are commonly comorbid with RLS include anxiety and depression, and our observations indicate that Chinese ALS patients with RLS symptoms has more serious anxiety than ALS/RLS– patients (32).

Diagnosis of RLS can be difficult in ALS patients because many conditions that mimic RLS should be excluded (13). There is a possibility that RLS symptoms in ALS patients were misdiagnosed and may be in fact a manifestation of ALS-related symptoms such as spasms or pain in limbs. However, the spasms or pain in ALS patients does not occur in the context of the circadian rhythm. In our study, the diagnostic criteria were well-defined and have previously been shown to be highly sensitive and specific (19). Furthermore, based on new diagnostic criteria and on our clinical experience, we designed an additional questionnaire to exclude mimicking symptoms in patients with ALS, thus giving further support to the diagnosis (18).

To date the available therapeutic for ALS is limited, riluzole, only slows the rate of progression and prolongs survival by 3 months (1). Therefore, great emphasis must be placed on alleviating symptoms and improving quality of life. At present, RLS symptoms, which occur frequently in ALS patients and can be effectively treated, remain underdiagnosed and undertreated (5, 9). The consequences of RLS can be severe, including disrupted nighttime sleep and increased depression, and may reduce the quality of life (29). Therefore, identification and management of RLS symptoms should be improved in ALS patients.

This study should be interpreted with consideration of several limitations. First, no genetic testing was performed in this study. However, the RLS patients in our study had no family history and, moreover, the role of genetic risk factors in secondary RLS patients seems to be rather limited (29). Second, the conclusions drawn from a single center-based observation could be influenced by referral bias, Some patients with fast progress might not come to our hospital and therefore these conclusions should be confirmed by further multicenter-based and population-based studies. Third, although we described the main clinical characteristics of RLS in ALS patients, the IRLSSG severity scale was not applied in our study. Despite the ease of use of the IRLSSG scale, it does not objectively consider motor dysfunction and psychological stress in ALS patients, and the outcome is highly dependent on a patient's degree of physical activity. Further, the IRLSSG scale is very sensitive to placebo effects (18). Fourth, we did not preform blood gas evaluation in the present study and we could not collect FVC data from a small proportion of ALS patients due to serious physical dysfunction or because they refused the examination, while polysomnographic data were available for only a few ALS patients, whereas the diagnosis of RLS is based on clinical (12). Moreover, PLMS could not excluded RLS mimic symptoms (9). Additional limitations of our study include that we did not evaluate the subclinical involvement of the thalamus in ALS/RLS+ patients, and we did not measure glutamate concentrations with PET or MRI in ALS/RLS+ patients. However, these methods are included in our ongoing work.

## Conclusion

we found a high frequency of RLS symptoms in Chinese ALS patients and these patients may benefit from recognition of this condition and from optimized management of its symptoms. Moreover, ALS patients might cause circadian rhythm disturbances and our study further supports that ALS is a heterogeneous disorder involving multiple systems; further studies are needed to confirm these preliminary findings.

## Author contributions

SL: study concept, data acquisition and statistical analyses, interpretation of the results, writing the first version of the manuscript; YH: study concept, RLS diagnosis, interpretation of the results, revising the manuscript; LC: study concept, ALS diagnosis, interpretation of the results, writing the final version of the manuscript; DS, HT, NS, HF, KZ, and ZW: revising the manuscript; QD: EMG study; ML: ALS diagnosis, revising the manuscript.

### Conflict of interest statement

The authors declare that the research was conducted in the absence of any commercial or financial relationships that could be construed as a potential conflict of interest.

## References

[1] Swinnen B Robberecht W. The phenotypic variability of amyotrophic lateral sclerosis. Nat Rev Neurol. (2014) 10:661–70. 10.1038/nrneurol.2014.184 25311585

[2] Ravits JM La Spada AR. ALS motor phenotype heterogeneity, focality, and spread: deconstructing motor neuron degeneration. Neurology (2009) 73:805–11. 10.1212/WNL.0b013e3181b6bbbd 19738176 PMC2739608

[3] Goldstein LH Abrahams S. Changes in cognition and behaviour in amyotrophic lateral sclerosis: nature of impairment and implications for assessment. Lancet Neurol. (2013) 12:368–80. 10.1016/S1474-4422(13)70026-7 23518330

[4] Braak H Brettschneider J Ludolph AC Lee VM Trojanowski JQ Del TK. Amyotrophic lateral s clerosis–a model of corticofugal axonal spread. Nat Rev Neurol. (2013) 9:708–14. 10.1038/nrneurol.2013.221 24217521 PMC3943211

[5] Lo Coco D Mattaliano P Spataro R Mattaliano A La Bella V. Sleep-wake disturbances in patients with amyotrophic lateral sclerosis. J Neurol Neurosurg Psychiatry (2011) 82:839–42. 10.1136/jnnp.2010.228007 21217159

[6] Cui B Cui L Gao J Liu M Li X Liu C. Cognitive impairment in chinese patients with sporadic amyotrophic lateral sclerosis. PLoS ONE (2015) 10:e137921. 10.1371/journal.pone.0137921 26367133 PMC4569418

[7] Ahmed RM Newcombe REA Piper AJ Lewis SJ Yee BJ Kiernan MC. Sleep disorders and respiratory function in amyotrophic lateral sclerosis. Sleep Med. Rev. (2015) 26:33–42. 10.1016/j.smrv.2015.05.007 26166297

[8] Dauvilliers Y Winkelmann J. Restless legs syndrome: update on pathogenesis. Curr Opin Pulmonary Med. (2013) 19:594–600. 10.1097/MCP.0b013e328365ab07 24048084

[9] Trenkwalder C Winkelmann J Inoue Y Paulus W. Restless legs syndrome-current therapies and management of augmentation. Nat Rev Neurol. (2015) 11:434–45. 10.1038/nrneurol.2015.122 26215616

[10] Azmin S Khairul AA Nafisah WY Tan HJ Raymond AA Hanita O. Restless legs syndrome and its associated risk factors in Parkinson's disease. Parkinsons Dis. (2013) 2013:535613. 10.1155/2013/535613 24455416 PMC3880760

[11] Peter-Derex L Yammine P Bastuji H Croisile B. Sleep and Alzheimer's disease. Sleep Med Rev. (2015) 19:29–38. 10.1016/j.smrv.2014.03.007 24846773

[12] Limousin N Blasco H Corcia P Arnulf I Praline J. The high frequency of restless legs syndrome in patients with amyotrophic lateral sclerosis. Amyotroph Lateral Scler. (2011) 12:303–6. 10.3109/17482968.2011.557736 21314298

[13] Lo Coco D Piccoli F La Bella V. Restless legs syndrome in patients with amyotrophic lateral sclerosis. Mov Disord. (2010) 25:2658–61. 10.1002/mds.23261 20669314

[14] Shi Y Yu H Ding D Yu P Wu D Hong Z. Prevalence and risk factors of restless legs syndrome among Chinese adults in a rural community of Shanghai in China. PLoS ONE (2015) 10:e0121215 10.1371/journal.pone.0121215 25803876 PMC4372383

[15] Panda S Gourie-Devi M Sharma A. Sleep disorders in amyotrophic lateral sclerosis: A questionnaire-based study from India. Neurol. India 66:700–8 (2018) 10.4103/0028-3886.232327 29766929

[16] Cedarbaum JM Stambler N Malta E Fuller C Hilt D Thurmond B. The ALSFRS-R: a revised ALS functional rating scale that incorporates assessments of respiratory function. BDNF ALS Study Group (Phase III). J Neurol Sci. (1999) 169:13–21. 10.1016/S0022-510X(99)00210-5 10540002

[17] Brooks BR Miller RG Swash M Munsat TL. El Escorial revisited: revised criteria for the diagnosis of amyotrophic lateral sclerosis. Amyotroph Lateral Scler Other Motor Neuron Disord. (2000) 1:293–9. 10.1080/146608200300079536 11464847

[18] Allen RP Picchietti D Hening WA Trenkwalder C Walters AS Montplaisi J. Restless legs syndrome: diagnostic criteria, special considerations, and epidemiology. A report from the restless legs syndrome diagnosis and epidemiology workshop at the National Institutes of Health. Sleep Med. (2003) 4:101–19. 10.1016/S1389-9457(03)00010-8 14592341

[19] Allen RP Picchietti DL Garcia-Borreguero D Ondo WG Walters AS Winkelman JW. Restless legs syndrome/Willis-Ekbom disease diagnostic criteria: updated International Restless Legs Syndrome Study Group (IRLSSG) consensus criteria–history, rationale, description, and significance. Sleep Med. (2014) 15:860–73. 10.1016/j.sleep.2014.03.025 25023924

[20] Buysse DJ Reynolds CR Monk TH Berman SR Kupfer DJ. The Pittsburgh Sleep Quality Index: a new instrument for psychiatric practice and research. Psychiatry Res. (1989) 28:193–213. 10.1016/0165-1781(89)90047-4 2748771

[21] Johns MW. A new method for measuring daytime sleepiness: the Epworth sleepiness scale. Sleep (1991) 14:540–5. 10.1093/sleep/14.6.540 1798888

[22] Stiasny-Kolster K Mayer G Schafer S Moller JC Heinzel-Gutenbrunner M Oertel WH. The REM sleep behavior disorder screening questionnaire–a new diagnostic instrument. Mov Disord. (2007) 22:2386–93. 10.1002/mds.21740 17894337

[23] Chen D Guo X Zheng Z Wei Q Song W Cao B. Depression and anxiety in amyotrophic lateral sclerosis: correlations between the distress of patients and caregivers. Muscle Nerve (2015) 51:353–7. 10.1002/mus.24325 24976369

[24] Allen RP Barker PB Horska A Earley CJ. Thalamic glutamate/glutamine in restless legs syndrome: increased and related to disturbed sleep. Neurology (2013) 80:2028–2034. 10.1212/WNL.0b013e318294b3f6 23624560 PMC3716406

[25] Kaur J Khararjian A Coleman RA Constantinescu CC Pan ML Mukherjee J. Spinal cord dopamine D2/D3 receptors: *in vivo* and *ex vivo* imaging in the rat using (18)F/(11)C-fallypride. Nucl. Med. Biol. (2014) 41:841–7. 10.1016/j.nucmedbio.2014.08.002 25199843 PMC4192035

[26] Baumann CR. Traumatic brain injury and disturbed sleep and wakefulness. Neuromole Med. (2012) 14:205–12. 10.1007/s12017-012-8178-x 22441999

[27] Tholfsen LK Larsen JP Schulz J Tysnes OB Gjerstad MD. Development of excessive daytime sleepiness in early Parkinson disease. Neurology (2015) 85:162–8. 10.1212/WNL.0000000000001737 26085603

[28] Pagano G Molloy S Bain PG Rabiner EA Chaudhuri KR Brooks DJ. Sleep problems and hypothalamic dopamine D3 receptor availability in Parkinson disease. Neurology (2016) 87:2451–6. 10.1212/WNL.0000000000003396 27807182 PMC5177673

[29] Garcia-Borreguero D Williams AM. An update on restless legs syndrome (Willis-Ekbom disease): clinical features, pathogenesis and treatment. Curr Opin Neurol. (2014) 27:493–501. 10.1097/WCO.0000000000000117 24978636

[30] Coulon P Budde T Pape HC. The sleep relay–the role of the thalamus in central and decentral sleep regulation. Pflugers Arch. (2012) 463:53–71. 10.1007/s00424-011-1014-6 21912835

[31] Chio A Pagani M Agosta F Calvo A Cistaro A Filippi M. Neuroimaging in amyotrophic lateral sclerosis: insights into structural and functional changes. Lancet Neurol. (2014) 13:1228–40. 10.1016/S1474-4422(14)70167-X 25453462

[32] Becker PM Sharon D. Mood disorders in restless legs syndrome (Willis-Ekbom disease). J Clin Psychiatry (2014) 75:e679–94. 10.4088/JCP.13r08692 25093484

